# *KCNN4* and *S100A14* act as predictors of recurrence in optimally debulked patients with serous ovarian cancer

**DOI:** 10.18632/oncotarget.9721

**Published:** 2016-05-30

**Authors:** Haiyue Zhao, Ensong Guo, Ting Hu, Qian Sun, Jianli Wu, Xingguang Lin, Danfeng Luo, Chaoyang Sun, Changyu Wang, Bo Zhou, Na Li, Meng Xia, Hao Lu, Li Meng, Xiaoyan Xu, Junbo Hu, Ding Ma, Gang Chen, Tao Zhu

**Affiliations:** ^1^ Cancer Biology Research Center (Key Laboratory of the Ministry Of Education), Tongji Hospital, Tongji Medical College, Huazhong University of Science and Technology, Wuhan, 430030, China

**Keywords:** serous ovarian cancer, recurrence, prognosis, *KCNN4*, *S100A14*

## Abstract

Approximately 50-75% of patients with serous ovarian carcinoma (SOC) experience recurrence within 18 months after first-line treatment. Current clinical indicators are inadequate for predicting the risk of recurrence. In this study, we used 7 publicly available microarray datasets to identify gene signatures related to recurrence in optimally debulked SOC patients, and validated their expressions in an independent clinic cohort of 127 patients using immunohistochemistry (IHC). We identified a two-gene signature including *KCNN4* and *S100A14* which was related to recurrence in optimally debulked SOC patients. Their mRNA expression levels were positively correlated and regulated by DNA copy number alterations (CNA) (*KCNN4*: *p=*1.918e-05) and DNA promotermethylation (*KCNN4*: *p=*0.0179; *S100A14*: *p=*2.787e-13). Recurrence prediction models built in the TCGA dataset based on *KCNN4* and *S100A14* individually and in combination showed good prediction performance in the other 6 datasets (AUC:0.5442-0.9524). The independent cohort supported the expression difference between SOC recurrences. Also, a KCNN4 and S100A14-centered protein interaction subnetwork was built from the STRING database, and the shortest regulation path between them, called the KCNN4-UBA52-KLF4-S100A14 axis, was identified. This discovery might facilitate individualized treatment of SOC.

## INTRODUCTION

Epithelial ovarian cancer (EOC) is the fifth leading cause of cancer-related death among women in the United States, with approximately 21,290 new patients and 14,180 deaths in 2015 [1]. Serous ovarian cancer (SOC) is the most common histological subtype of EOC, accounting for 87% of advanced stage cases and 78% of total cases [2]. To date, the gold standard treatment for ovarian cancer involves cytoreductive surgery followed by platinum- and paclitaxel-based chemotherapy. Although a substantial proportion of individuals achieve a complete clinical response [3, 4], 50-75% of the patients suffer relapse within 18 months after first-line treatment [3], which means that SOC are scarcely ever curable, and generally have short-term progression-free survival [5]. In brief, recurrence is the first critical clinical episode that has a significant impact on prognosis. Recurrence under the premise of optimal debulking reflects the essence of tumor heterogeneity more than external factors. Nevertheless, with only clinical indicators, such as patient age, tumor stage, tumor grade and so on, it is difficult to predict which SOC patients will develop recurrence after optimal debulking surgery and standard first-line chemotherapy.

The development of high-throughput techniques, such as microarray and sequencing, will be helpful to gain insight into the molecular profiles of tumor cells in the context of recurrence. For the past few years, several studies have applied gene expression profiling to ovarian cancer [6–11]. However, only a small number of genes were shared among these gene profiles identified in separate studies [12]. No robust prediction models on recurrence or overall survival have been established. It is urgent to establish novel and pragmatic gene signatures robustly applicable in multiple datasets with a large number of cases.

In this study, we strived to excavate novel gene signatures for recurrence in patients with SOC under the premise of optimal debulking surgery. We discovered 2 candidate gene markers, *KCNN4* and *S100A14*, relevant for recurrence from 7 publicly available microarray datasets. Recurrence forecasting models based on the 2 genes performed well. Consistent with our study, prior studies demonstrated that *S100A14* was overexpressed in SOC at both mRNA and protein levels [13]. To our knowledge, there is little valuable information on the role of *KCNN4* in ovarian cancer or other cancers [14, 15]. This is the first report indicating that the *KCNN4* gene acts as an independent predictor for recurrence in SOC patients and also may be a very attractive therapeutic target. Also, the KCNN4-UBA52-KLF4-S100A14 axis we constructed may be the potential regulatory pathway between KCNN4 and S100A14 proteins, representing a novel regulatory mode impacting the early prognosis of SOC. Finally, we examined the protein expressions of KCNN4 and S100A14 in an independent clinical cohort of 127 SOC patients, which also supported the conclusion drawn from these 7 public datasets that these genes could be independent prognostic factors for recurrence in optimally debulked SOC patients.

## RESULTS

### Dataset characteristics and the identification of genes associated with an increased incidence of recurrence

After rigorous screening, 7 datasets (TCGA [16], TCGA.RNASeqV2 [16], GSE17260 [17], GSE26193 [18], GSE30161 [19], GSE49997 [20], and GSE9891 [21]) were obtained from the *curatedOvarianData* package according to the screening criteria detailed in Methods. In these 7 datasets, 305, 156, 37, 77, 17, 120, and 126 samples, respectively, met the inclusion criteria for our analysis. The general characteristics of the 7 datasets are listed in Table 1. In these datasets, patients who received optimal debulking surgery and relapsed after more than 90 days from the termination of first-line treatments were classified into two groups according to their recurrence status (recurrence versus no recurrence). The top 2000 genes were selected with a signal-to-noise ratio (SNR) criteria for the 7 datasets, and the common genes obtained via intersection were exactly *KCNN4* and *S100A14*.

**Table 1:** general information of involved 7 public datasets

Datasets	Platform	Sample number	Screened Samples^*^	Age (year)^#^ median (range)	Recurrence status^#^		Days to recurrence^#^ median (range)	Vital status^#^		Days to death^#^ Median (range)
					no recurrence	recurrence		deceased	living	
TCGA	hthgu133a	481	305	57 (30 - 84)	131	174	453 (92 - 3378)	156	149	981 (92 - 4623)
TCGA RNASeq	RNASeqV2	242	156	56 (34 - 84)	68	88	423 (92 - 2648)	83	73	913 (92 - 4623)
GSE17260	hgug4112a	84	37	−	19	18	690 (120 - 2250)	10	27	990 (270 - 2250)
GSE26193	hgu133plus2	79	77	−	16	61	595 (121 – 7386)	58	19	1136 (194 - 7386)
GSE30161	hgu133plus2	45	17	55 (47 - 75)	3	14	566 (162 - 4208)	9	8	1846 (377 - 4208)
GSE49997	ABI	171	120	56 (27 - 85)	51	69	533 (122 - 1461)	30	90	776 (122 - 1491)
GSE9891	hgu133plus2	239	126	59.5 (39 - 80)	42	84	540 (120 - 3060)	50	76	900 (180 - 6420)

* Samples screened according to the criteria mentioned in the method section: optimal debulking and days_to_tumor_recurrence > 90d.

# summarized according to screened samples.

### Correlation analysis of *KCNN4* and *S100A14*

**Figure 1:**
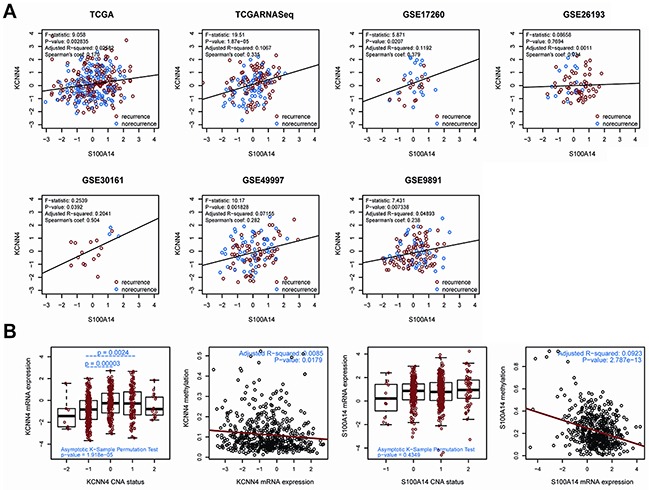
**A.** The correlation of gene expression between *KCNN4* and *S100A14* in 7 public datasets. In each dataset, recurrent samples and norecurrent samples were marked in red and blue, respectively. Some statistical results are also listed. The black solid line represents the linear regression; **B.** the correlation of the expression profiles of KCNN4 and S100A14 with CNA status as well as methylation values. For CNA status, −2 = homozygous deletion, −1 = hemizygous deletion, 0 = neutral/no change, 1 = gain, and 2 = high level amplification. The total significance was estimated from the null distribution constructed by the asymptotic K-sample permutation test. If significant, pairwise comparisons were then performed via TukeyHSD test, and the *p* values were adjusted with the BH method. The *p* values are also labeled. For methylation status, the red lines represent the linear regression between the expression values and methylation values.

Through linear regression analysis, we preliminarily explored the linear relationship between the expression of *KCNN4* and *S100A14* in SOC, and the mRNA expressions of *KCNN4* and *S100A14* were significantly positively correlated in 6 datasets (*p*<0.05) except in the GSE26193 dataset (*p*=0.7694) (Figure 1A). The expression distributions of *KCNN4* and *S100A14* are shown based on recurrence status with dot and density plots in the 7 datasets (Supplementary Figure S1). Nearly half (42.9% (6/14)) of these expression distributions showed significant differences between the recurrence statuses (*p*=0.00473~0.4195). Furthermore, for detecting possible regulation mechanisms associated with the *KCNN4* and *S100A14* expression values, we measured the correlations of the mRNA expression values of *KCNN4* and *S100A14* with their respective copy number alterations (CNA) as well as DNA methylation (Figure 1B). The copy number aberrations had 5 distinct statuses: homozygous deletion (−2), hemizygous deletion (−1), neutral (no change, 0), gain (+1), and high level amplification (+2). CNA status was significantly positively correlated with mRNA expression values of *KCNN4* (*p*=1.918e-05). Moreover, we found a significant expression difference between the hemizygous deletion (−1) and neutral (0) (*p*=0.00003) statuses, as well as between the hemizygous deletion (−1) and gain (+1) (*p*=0.0024) statuses. However, no statistical significance was observed between the CNA status and the mRNA expression values of *S100A14* (*p*=0.4349). Both *KCNN4* and *S100A14* had significant negative correlations between their mRNA expression values and DNA methylation values (*p*=0.0179 and 2.787e-13, respectively).

### *KCNN4* and *S100A14* act as independent predictors of recurrence

**Figure 2:**
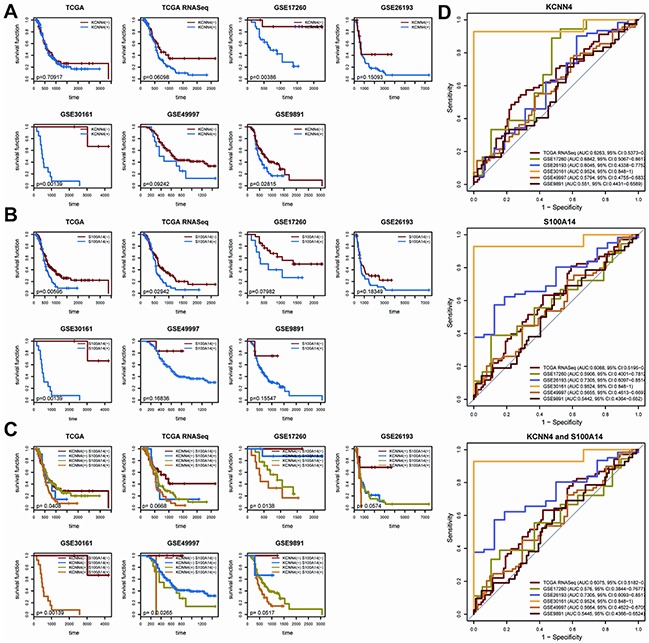
**A.** The KM plot of recurrence with different *KCNN4* expression states. (−) means lower expression and (+) means higher expression; **B.** The KM plot of recurrence with different *S100A14* expression states; **C.** The KM plot of recurrence with combined *KCNN4* and *S100A14* states; **D.** The prediction power of the recurrence prediction model built with TCGA dataset in the other 6 datasets. The model was built with linear kernel SVM via 5-repeats of 10-fold cross validation, coupled with internal parameter selection procedures. For each dataset, the AUCs and 95%CIs are also listed.

We determined the expression cut-off points of *KCNN4* and *S100A14* in The Cancer Genome Atlas (TCGA) dataset as described in Methods, and their expression levels were classified as “low”(−) and “high”(+) accordingly. The *KCNN4* expression status, *S100A14* expression status, and their crossed combination status were significantly correlated with recurrence status in 7 datasets, and their odds ratios (ORs), 95% confidence indexes (CI), and *p* values are listed (Table 2). Higher mRNA expressions of *KCNN4* or *S100A14* were significantly correlated with higher recurrence rates of optimal debulked SOC patients in all datasets. Nevertheless, more significant expression difference were obtained by combining their expression statuses to form 4 crossed groups, namely, *KCNN4*(−)*S100A14*(−), *KCNN4*(+)*S100A14*(−), *KCNN4*(−)*S100A14*(+), and *KCNN4*(+)*S100A14*(+). The expression difference according to the 4 crossed groups seemed to be more significant than those of 2 combined groups in which *KCNN4*(−)*S100A14*(−) was compared with the other 3 statuses. Furthermore, statistical analysis of the relationships of the various clinical factors and their mRNA expression levels/statuses was systematically performed for the 7 datasets, and there were no obviously significant relationships among them except for recurrence status (Supplementary Table S1). According to our statistical outcomes, the *KCNN4* expression status were significantly correlated with recurrence in 5 datasets (TCGA, TCGA.RNASeqV2, GSE17260, GES26193 and GSE30161), and the *KCNN4* expression values were significantly correlated with recurrence in 2 datasets (TCGA.RNASeqV2 and GSE30161). Both of the *S100A14* expression status and the *S100A14* expression values were correlated with recurrence in the same 4 datasets (TCGA, TCGA.RNASeqV2, GSE26193, and GSE30161). As shown in Figure 2A-2C, the Kaplan-Meier curves and log-rank tests emphasized the classification abilities on recurrence of the univariate expression statuses and combined expression status. It is noteworthy that, because they were initially screened according to differential expression on recurrence status, we could classify overall survival based on them in some datasets, but the results were not as robust as observed upon recurrence (Supplementary Figure S2). Moreover, according to the univariate and multivariate Cox regression analyses, the *S100A14* and *KCNN4* mRNA expression levels tended to be independently correlated with recurrence in the majority of datasets, while the *KCNN4* mRNA expression level might serve as an independent prognostic factor for overall survival in the TCGA and GSE17260 datasets, as well as *S100A14* expression levels in the GSE26193 and GSE49997 datasets (Supplementary Table S2). These results suggest that there were significant expression differences in early prognosis rather than late prognosis between the mRNA expression statuses of *KCNN4* and *S100A14*. Thus, these genes could be employed as recurrence predictors and were not suitable for the prediction of overall survival. Prediction models were built in the TCGA dataset on the basis of *KCNN4* and *S100A14* mRNA expression values, as well as their combination, using linear kernel SVM (svmLinear) to explore their prediction power for recurrence (Figure 2D). The area under ROC curve(AUC) and 95% confidence intervals (CI) of these 3 models showed good performance in the other 6 datasets (the highest AUC: 0.9524, 95%CI: 0.848-1; the lowest AUC: 0.5442, 95%CI: 0.4364-0.652). Additional 9 models were built by 3 other machine learning algorithms: random forest (rf), radial kernel SVM (svmRadial), and the artificial neural network (nnet), and the AUC and 95%CIs of these models are also presented (Supplementary Figure S3). Basically, these models had prediction abilities consistent with the 3 models built by linear SVM, which means that the mRNA expression levels of *KCNN4* and *S100A14* had a stable prediction power for recurrence, regardless of which machine learning algorithm was adopted. From the above results, we can conclude that the mRNA expressions of *KCNN4* and *S100A14* are independent prognostic indicators for recurrence in optimal debulked SOC patients.

**Table 2:** Expression difference of *KCNN4* and *S100A14* on SOC recurrence

Dataset	*KCNN4*			*S100A14*			*KCNN4+S100A14*			
	OR	95%CI	P	OR	95%CI	P	OR	95%CI	P^a^	P^b^
**TCGA**	1.79	1.08 – 2.98	**0.0206**	2.12	1.13 – 4.13	**0.0146**	1.87	1.11 – 3.18	**0.0161**	**0.0117**
**TCGA RNASeq**	3.18	1.52 – 6.79	**0.0011**	2.91	1.33 - 6.68	**0.0051**	4.13	1.87 - 9.51	**0.0001**	**0.0005**
**GSE17260**	14.23	1.58 – 705.97	**0.0078**	3.28	0.59 – 24.07	0.151	3.28	0.59 – 24.07	0.151	**0.0165**
**GSE26193**	5.34	1.17 – 24.84	**0.0142**	9.18	1.86 – 90.23	**0.0016**	1.53	0.44 – 5.41	0.5704	**0.0004**
**GSE30161**	Inf	1.98 – Inf	**0.0059**	Inf	1.98 – Inf	**0.0059**	Inf	1.98 – Inf	**0.0059**	**0.0059**
**GSE49997**	4.11	0.82 – 40.35	0.0691	7.28	0.78 – 353.95	0.0821	Inf	0.02 - Inf	1	**0.0068**
**GSE9891**	1.82	0.76 – 4.58	0.1689	6.28	0.49 – 338.42	0.1075	2.06	0.85 – 5.35	0.111	0.0878

a This comparison were done between KCNN4(−)S100A14(−) and KCNN4(+)S100A14(+)/KCNN(+)S100A14(−)/KCNN(−)S100A14(+)

b Comparisons among 4 groups such as KCNN4(−)S100A14(−), KCNN4(+)S100A14(+), KCNN(+)S100A14(−) and KCNN(−)S100A14(+)

### A KCNN4- and S100A14-centered interaction subnetwork

**Figure 3:**
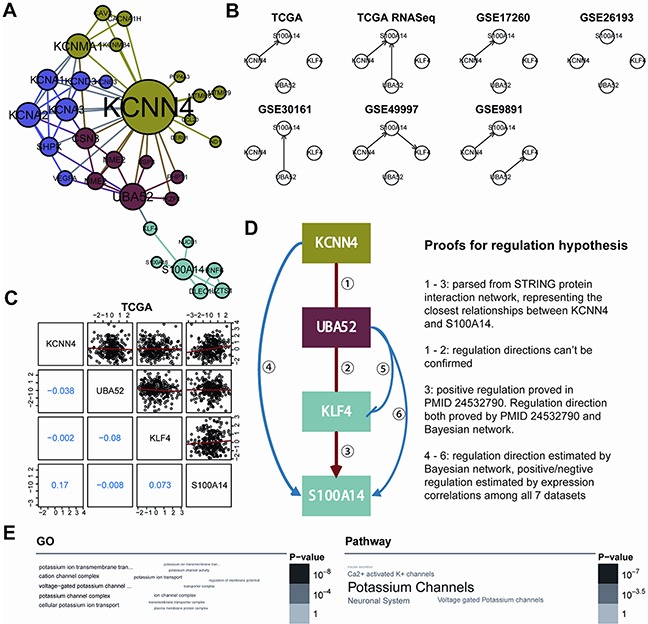
**A.** The KCNN4 and S100A14 centered interaction subnetwork. The minimum network connected KCNN4 and S100A14 constructed from a high quality STRING database (combined score >= 600) when restricted to 1-NN of KCNN4 and S100A14. Four communities were detected using fast greedy searching and are colored differently. The node sizes are proportional to the degrees of each gene; **B.** The regulation frameworks of the KCNN4-UBA52-KLF4-S100A14 axis determined by the Bayesian network based on hill-climbing scoring. The arrows are the regulation directions; **C.** Pairwise correlations of expression profiles of 4 genes in the TCGA dataset. The upper triangle showed the paired expression in all TCGA samples and the red lines represent the linear regression results. The lower triangle illustrated the pairwise Pearson's correlation coefficients; **D.** Hypothesized regulation modes of the KCNN4-UBA52-KLF4-S100A14 axis. The frames were colored according to the colors of the communities to which they belong. The arrows mean stimulation and the blocked arrow means inhibition. To the right is the proof for the regulation hypothesis; **E.** Word cloud representation of enriched GO and pathway terms on genes in the subnetwork (adjusted p<0.05). Their significance are illustrated with different font size and gray scale.

To further interpret the interaction relationship between *KCNN4* and *S100A14*, we built the minimal KCNN4 and S100A14-centered undirected interaction subnetwork from the high quality STRING protein interaction database (combined score ≥ 600) with k nearest neighbors (k=1) (Figure 3A). The shortest path between KCNN4 and S100A14, namely, the KCNN4-UBA52-KLF4-S100A14 axis, was determined. We first speculated the most probable regulation directions using Bayesian network inference based on the hill-climbing algorithm and evaluated their performance with Akaike information criterion (AIC). Intriguingly, the regulatory directions were also almost consistent in the 7 datasets, and *S100A14* was always regulated by *KCNN4* (Figure 3B). We then measured the pairwise expression correlations of these 4 genes in the 7 datasets (Figure 3C; Supplementary Figure S4), and some consistent negative/positive correlations were identified. Combining the above results, the final hypothetical regulation mode of this axis is shown as well as the relevant proofs (Figure 3D). Because the pairwise interactions were derived from convincing benchwork results and we had confirmed the consistent positive correlations of KCNN4 and S100A14 in all 7 datasets, we speculated that this axis might represent the most direct regulatory pathway between KCNN4 and S100A14 in SOC. In detail, in the axis, the regulation directions tended to flow from KCNN4 to S100A14. The directions between KCNN4 and UBA52, and between UBA52 and KLF4, could not be determined by Bayesian network inference and had no solid support from other traceable resources. The regulation direction between KLF4 and S100A14 was also demonstrated in breast cancer [22], as was the inference from our Bayesian network. The promotion of S100A14 on KCNN4 and UBA52 stimulation, as well as the inhibition of KLF4 by UBA52, was supported by Bayesian network inference. The stimulation and inhibition causality were confirmed by pairwise expression correlations throughout all 7 datasets. Therefore, with the support of Bayesian network inference, pairwise expression correlations, and previous research conclusions, we gained sufficient confidence about the reliability of the hypothetical regulation mode of this axis. Furthermore, we detected the KCNN4 and S100A14 centered subnetwork in the community with a fast greedy algorithm [23], and 4 distinct communities were detected and colored accordingly. We found that these 4 axis genes belonged to 3 communities, that KCNN4, UBA52 and S100A14 were hub genes in their own communities, and that KLF4 was a key connector between UBA25 and S100A14. GO and pathway enrichment analysis of the minimal subnetwork showed that it was primarily involved in potassium ion transport (Figure 3E).

### KCNN4 and S100A14 protein expression in SOC assessed by IHC

**Figure 4:**
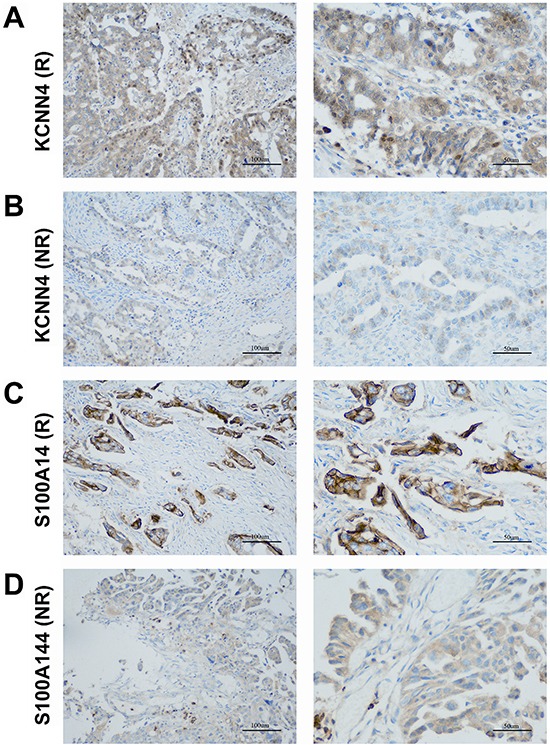
Representative patterns of KCNN4 and S100A14 IHC staining in the SOC cohort **A.** KCNN4 staining in SOCs with recurrence (R); **B.** KCNN4 staining in SOCs with norecurrence (NR); **C.** S100A14 staining in SOCs with recurrence (R); **D.** S100A14 staining in SOCs with norecurrence (NR). Original magnification: ×200 and ×400. Scale bars, 100 μm, 50 μm.

**Figure 5:**
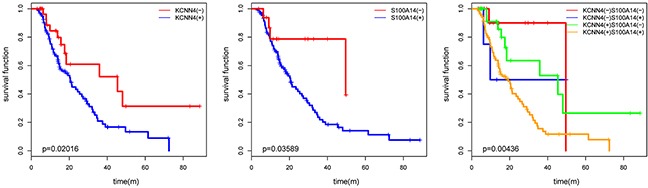
The KM plot of SOC recurrence with KCNN4 and S100A14 expression status in our cohort The *p* values from log-rank tests are also presented.

We examined the protein expression of KCNN4 and S100A14 in an independent SOC cohort using immunohistochemistry (IHC) staining. KCNN4-positive staining was predominantly localized in the nucleus and cytoplasm of cancer cells. S100A14-positive staining was mainly localized to the cytoplasm and cytomembrane in cancer cells. Representative IHC KCNN4 and S100A14 staining shapes are shown in Figure 4. The main clinicopathologic parameters of 127 SOC patients in our independent cohort underwent optimal debulking surgery, and their relationships with the IHC expression of KCNN4 and S100A14 are shown (Table 3). Significant differences in both the KCNN4 and S100A14 IHC expressions were found between recurrent cases and norecurrent cases (*p*=0.02301 and *p*=0.002346, respectively). As for the recurrence distribution between the KCNN4 and S100A14 combined groups, higher significance was achieved (*p*=3.607e-06). Additionally, high KCNN4 IHC expression was associated with FIGO stage (*p*=0.002668), and high S100A14 IHC expression was significantly associated with positive lymph node metastasis (*p*=0.02569). According to the univariate and multivariate Cox regression analyses, both KCNN4 and S100A14 IHC expressions were correlated with recurrence, so KCNN4 and S100A14 could also be established as independent predictors for SOC recurrence within our cohort (Table 4). Kaplan-Meier curves were also plotted, and log-rank tests were performed to compare the recurrence predictions of singular KCNN4 and S100A14 IHC expression in our cohort. In all situations, there were significant differences among the different IHC expression levels, and their combined IHC groups seemed to have better prediction abilities than the single genes, as illustrated in the above public SOC datasets (KCNN4: *p*=0.02016; S100A14: *p*=0.03589; KCNN4 and S100A14: *p*=0.00436) (Figure 5).

**Table 3:** Relationship of *KCNN4* and *S100A14* expression with clinicopathological characteristics in SOC cohort

clinicopathological characteristics	No.	KCNN4			S100A14		
		high(+)	χ^2^	p value	high(+)	χ^2^	p value
**Age(years)**							
≥60	36	25(69.4%)	0.7655	0.3816	31(86.1%)	0.0110	0.9166
<60	91	70(76.9%)			79(86.8%)		
**Stage**							
I~II	37	21(56.8%)	9.0215	**0.002668**	33(89.2%)	0.2986	0.5848
III~IV	90	74(82.2%)			77(85.6%)		
**Grade**							
Grade1	8	5(62.5%)	0.692	0.7075	7(87.5%)	4.6933	0.0957
Grade2	24	18(75.0%)			24(100%)		
Grade3	95	72(75.8%)			79(83.2%)		
**CA125(U/ml)**							
≥35	32	25(78.1%)	0.2505	0.6168	24(75.0%)	4.9771	**0.02569**
<35	95	70(73.7%)			86(90.5%)		
**Ascites**							
Positive	77	54(70.1%)	2.2662	0.1322	67(95.7%)	0.02683	0.8699
Negative	50	41(82.0%)			43(86.0%)		
**Tumor size(cm)**							
≥5	87	66(75.9%)	0.1643	0.6852	72(82.8%)	0.9981	0.3178
<5	40	29(72.5%)			38(95.0%)		
**Lymph node metastasis**							
Positive	26	19(73.1%)	0.0517	0.8202	19(73.1%)	5.1675	**0.02301**
Negative	101	76(75.2%)			91(90.1%)		
**Recurrence status**							
Positive	73	62(84.9%)	9.3443	**0.002237**	69(94.5%)	9.2566	**0.002346**
Negative	54	33(61.1%)			41(75.9%)		

**Table 4:** Univariate and multivariate Cox regression on recurrence in SOC cohort

Clinical factors	Univariate analysis			Multivariate analysis		
	OR	95%CI	Pr(>|z|)	OR	95%CI	Pr(>|z|)
**Age(≥60y/<60y)**	0.9994	0.7791 - 1.282	0.996	1.0227	0.7903 – 1.3234	0.8646
**Stage(I-II/III-IV)**	1.279	0.7905 - 2.068	0.316	1.1443	0.6615 – 1.9792	0.6298
**Grade(I/II/III)**	0.8208	0.5856 - 1.15	0.252	0.7624	0.5155 – 1.1276	0.1743
**CA125(≥35/<35U/ml)**	1.081	0.8237 - 1.42	0.573	0.9872	0.7233 – 1.3473	0.9345
**Ascites(+/−)**	0.9758	0.7704 - 1.236	0.839	1.0479	0.8181 – 1.3423	0.7109
**Tumor size(≥5/<5cm)**	0.9477	0.745 - 1.206	0.662	0.8903	0.6716 – 1.1802	0.4192
**Lymph node metastasis(+/−)**	1.188	0.9086 - 1.553	0.208	1.1910	0.8922 – 1.5900	0.2356
**KCNN4(low/high)**	0.4711	0.2461 - 0.9018	**0.0231**	0.4807	0.2402 – 0.9623	**0.0386**
**S100A14(low/high)**	0.3551	0.1292 - 0.9755	**0.0446**	0.2955	0.1020 – 0.8566	**0.0248**

## DISCUSSION

During the development of ovarian cancer, recurrence is the first critical clinical episode that has significant influence on late prognosis. The identification of patients who may experience recurrence before first-line treatment would greatly benefit clinical management. Clinical factors have poor predictive power for recurrence. Until now, transcription profiling has been used to derive molecular signatures associated with the prognosis of ovarian cancer [6–11]. However, the gene signatures identified in these studies shared only a small number of genes [12]. The vast majority of previous researches had focused overall survival rather than initial recurrence. Nevertheless, the limited independent cohort datasets and small sample sizes in some studies raised doubts about the generalizability and creditability of these results. Furthermore, multigene signatures are not very convenient to apply in clinical routines, whether as clinical predicting indicators or as therapeutic targets. In our work, we identified that the high expression of *KCNN4* and *S100A14* was associated with a high incidence of recurrence in optimally debulked SOC patients on both the mRNA and protein levels. To our knowledge, this is the first report about the relationship between *KCNN4* and SOC. We established this 2-gene signature as an independent predictor for recurrence on optimally debulked SOC patients, robustly applicable in both multiple public datasets and our cohort data, and at both the mRNA and protein levels.

The *KCNN4* gene encodes a KCa3.1 channel. The KCa3.1 channel is an intermediate conductance Ca2+-activated potassium channel that is sensitive to changes in intracellular Ca2+ and is voltage-independent. It had been found to be related to tumor grade, cell proliferation, metastatic spread, and cell cycle progression in several cancer types including prostate, pancreas, and breast cancer [14, 24–27], melanoma [28], endometrial cancer [29], as well as non-small cell lung cancer (NSCLC) [30], but no reports can be seen on SOC. The KCa3.1 channel-specific inhibitor, senicapoc, has been shown to prevent NSCLC proliferation [30].

S100A14 protein is a type of EF-hand calcium-binding protein that can function both intracellularly and extracellularly. It is reported to be upregulated in some cancer types, including ovarian, lung, breast, uterine, and cervical cancer [31], but downregulated in others, for instance, kidney, colon, rectal, and esophageal cancer [32]. The overexpression of S100A14 is related with poor prognoses in breast cancer, liver cancer, cervical cancer and ovarian cancer [13, 31, 33–36] but is associated with favorable outcomes in colorectal and small intestinal cancers [37, 38]. Specifically for ovarian cancer, the prior studies of S100A14 on SOC demonstrated that S100A14 was overexpressed in transformed cells on both the RNA and protein levels, which is consistent with our results. They found that S100A14 expression is associated with advanced stage (*p*< 0.001) and poor tumor grade (*p*< 0.001), but this conclusion was neither universal in all public datasets nor could it be replicated with our cohort data. Their assertion that S100A14 overexpression was an independent prognostic factor for overall survival (HR=4.53, *p*=0.029) could be partially supported by the enrolled public datasets and our data. Based on their results, we found that S100A14 could also be an independent prognosis predictor for recurrence and achieved higher prediction power when combined with KCNN4.

Moreover, we discussed their positive correlations of gene expression, and genomic regulations, with promoter methylation and CNV. We also sponsored a KCNN4 and S100A14-centered subnetwork and extracted the shortest regulation path between them, which was called the KCNN4-UBA52-KLF4-S100A14 axis, and speculated convincible regulation modes among them with the aid of Bayesian network inference, pairwise expression correlations and prior study conclusions. GO and pathway analyses of this subnetwork show that it is primarily involved in potassium ion transport, and the axis may play a central role. Previous studies have also noted that potassium ion channels are related to the proliferation, apoptosis and drug resistance of ovarian cancer cells [39–44]. Because S100A14 is an EF-hand calcium-binding protein that has been reported to be involved in the progression of SOC, and KCa3.1 channel is a Ca2+-dependent potassium channel, we assumed there was some type of regulatory causality between these two genes in SOC. More daringly, we speculated that the recurrence of SOC is associated with potassium ion transport. Our future work will further validate the prognostic value of *KCNN4* and *S100A14*, as well as the regulation hypothesis of the axis, in additional to multicenter prospective studies of optimal debulked SOC patients.

## MATERIALS AND METHODS

### Public datasets for exploratory studies

All statistical analyses and data mining procedures in this work were carried out with R (version: 3.2.2). The source codes are available upon request. Bioconductor package *curatedOvarianData* (version: 1.0.5) introduced a manually curated data collection for gene expression meta-analysis of patients with ovarian cancer and software for reproducible preparation of similar databases, providing a comprehensive and flexible resource for clinically oriented investigation of the ovarian cancer transcriptome [45]. We employed an R script called createEsetList which was provided in this package to filter datasets and samples. The screening procedure was based on the following criteria: a. only keep optimal debulked SOC cases; b. should have recurrence related clinical data such as “recurrence status” and “time to tumor recurrence”; c. exclude cases with recurrence time less than 90 days form the termination of first-line therapies in order to avoid ambiguous outcomes; d. only keep common genes in all datasets; e. removed low correlation samples and duplicated samples. According to above criteria, 7 datasets were kept for further analysis. Finally, expression values in all datasets were normalized as standard Z-score. The clinical data of 7 datasets were listed in Table 1.

### Cohort specimens for IHC

Paraffin-embedded cancer tissue samples were obtained from 127 SOC patients who underwent optimal debulking surgery at Tongji Hospital from February 2007 to May 2014. Optimal debulking surgery was defined as residual disease ≤1 cm after initial cytoreductive procedure. Postoperative Platinum and Taxol based chemotherapies commonly carried out for 4-8 cycles. The study was approved by the Ethical Committee of the Medical Faculty of Tongji Medical College, and written informed consents were obtained from all patients included. Individuals with preoperative chemotherapy or radiotherapy upon recurrence were excluded from this study. Ultimately, a cohort of 127 patients, who meet standards all above, was selected for our study. The stage of tumors were evaluated based on the InternationalFederation of Gynecology and Obstetrics (FIGO) criteria, while tumor grade were determined according to World Health Organization (WHO) standards. All subjects were reconfirmed for diagnosis by two independent pathologists in a blind manner. The original clinical data were obtained from clinical records database, including age at surgery, tumor stage and grade, serum CA-125 level, tumor size, ascites, lymph node metastasis, recurrence status and so on. Detailed characteristics of all cases are summarized in Table 3. The follow-up period was calculated from the termination date of the first-line therapy to the date of last follow-up evaluation or recurrence. The duration of follow-up time ranged from 3 to 89 months, the median time was 14 months. 73 (57.4%) of these cases relapsed during the follow-up period.

### Recurrence related genes screening

We selected recurrence related genes by signal-to-noise (SNR) ratio between 2 recurrence status, that is, recurrence and norecurrence, in all these 7 ovarian cancer datasets. SNR is defined as |(μ_1_-μ_2_)/(σ_1_+σ_2_)|, in which μ_1_ and μ_2_ are mean values in two recurrence status, and σ_1_and σ_2_ are standard deviations in each group. Top 2000 genes which had high SNR in each dataset were selected and 2 intersected genes finally obtained, which were *KCNN4* and *S100A14*.

### Expression correlation between *KCNN4* and *S100A14*

To explore the mRNA expression correlation between *KCNN4* and *S100A14*, we made linear regressions between *their* expression in all 7 datasets. Their expression levels and distributions in all 7 datasets were also explored. To uncover the relationships of their mRNA expression with copy number alterations (CNA) and methylation, we extracted matched TCGA mRNA expression data, CNA data and methylation data of *KCNN4* and *S100A14* from cBioportal via *cgdsr* package [46, 47]. We estimated mRNA expression difference of *KCNN4* and *S100A14* among different CNA levels from a null distribution which was respectively constructed by asymptotic K-sample permutation test [48]. TukeyHSD tests were performed to detect pairwise significance, and all pairwise *p* values were adjusted with the method of Benjamini and Hochberg (BH). The linear regression of mRNA expression and methylation values were also performed.

### Survival analysis of *KCNN4* and *S100A14*

Since we screened these 2 targets from intersected recurrence status related genes, we wondered if they have prediction powers in early and late prognosis according to their mRNA expression levels. We decided their expression cutpoints in TCGA dataset to define binary expression status according to MinPvalue criterion from *OptimalCutpoints* package which aimed to minimize *p* value generated from χ2 test when measuring the association between the binary results obtained on using the cutpoint [49]. *KCNN4* and *S100A14* expression status were finally classified into “low” and “high” according to the comparisons between expression values with established cut-offs. The odds ratio (OR), 95% confidence index (CI), and *p* values of correlations between recurrence status and *KCNN4*, *S100A14* expression status as well as their combination were determined via Fisher's exact tests. Kaplan-Meier curves were drawn in univariate expression status as well as crossed expression status on recurrence, and log-rank tests were performed to check their significance. In order to check their classification abilities on late prognosis, we also performed log-rank tests on overall survival. We also explored the correlation of either *KCNN4*, *S100A14* expression values or status with various clinical factors such as stage, grade, age, and so on. According to different data types and statistical purposes, different statistical methods were adopted, which were labeled as table legends. At last, univariate and multivariate Cox regressions concerning clinical factors and *KCNN4* and *S100A14* expression status both in all 7 datasets and our cohort were performed.

### Recurrence prediction models construction and validation

To explore the prediction powers of *KCNN4* and *S100A14* expression on binary recurrence status, we constructed prediction models on TCGA dataset with 4 different machine learning algorithms, that is, random forest (rf), linear kernel support vector machine (svmLinear), radial kernel support vector machine (svmRadial), artificial neural network (nnet) using *caret* package [50]. The training models were built upon *KCNN4*, *S100A14* and both of them respectively. These training models were built via 5-repeats of 10-fold cross validations based on above algorithms and coupled with internal parameter selection procedures. The prediction performance were judged with ROC and kappa values originated from cross validation processes and the best models confirmed. These models were then validated in other 6 datasets.

### KCNN4 and S100A14 centered interaction subnetwork

To further investigate the regulation modes between KCNN4 and S100A14, we constructed KCNN4 and S100A14 centered interaction subnetwork from high quality STRING protein interaction database (combined score ≥ 600) [51]. We built the minimal undirected interaction subnetwork that could connect KCNN4 and S100A14 with k nearest neighbors (k=1). We found KCNN4 and S100A14 were connected by other 2 genes, UBA52 and KLF4. We called the shortest path between them as KCNN4-UBA52-KLF4-S100A14 axis which may represent the most potential and effective regulatory path. In order to investigate the potential regulation modes in this axis, we should confirm the positive or negative regulation modes and upstream or downstream regulation directions. To achieve these purposes, we further calculated pairwise correlation of expression values of axis genes. Then we determined the most appropriate regulation directions among axis genes by constructing Bayesian networks on axis genes in all 7 datasets with hill climbing method, which is a score-based learning algorithm and its conditional independent test is based on AIC [52], then their prediction results were combined. We employed fast greedy searching community detection algorithm on KCNN4 and S100A14 centered interaction subnetwork to find functional modules, and put the axis forward as a regulation hypothesis. Enrichment analysis of all genes involved in KCNN4 and S100A14 centered interaction subnetwork was performed by hypergeometric tests on GO and pathway database such as KEGG and REACTOME, and the significant enriched terms (adjusted p<0.05) were illustrated in word clouds [53].

### Immunohistochemistry analysis

IHC analysis was performed as described previously [54]. The treated tissue sections were incubated with rabbit anti-KCNN4 antibody (ABclonal, 1:50) and rabbit anti-S100A14 antibody (Proteintech, 1:100). IHC was performed via the G1210 kit (Wu hanGoodbio technology CO., LTD, Wuhan, China) according to manufacturer's instructions. Antibody binding was visualized by using 3, 3′-diaminobenzidine (DAB). The nuclei were counterstained with haematoxylin. Human tonsil and colon cancer tissues were selected as positive-control samples according to the manufacturers' instructions and included in each batch. Negative-control samples were processed as per the standard protocol but with the IgG antibody. All slides were scanned by OLYMPUS (DP73) scanning system. IHC staining of KCNN4 or S100A14 was semi-quantitatively score don the basis of positively stained area and staining intensity by two independent pathologists in a blinded manner. Staining intensity was scored as follows: 0 (negative), 1 (weak), 2 (moderate) and 3 (strong). While the percentage of positive cells wasalso scored as follows, 1(0-25%), 2(26-50%), 3(51-75%), 4 (76-100%). The levels of KCNN4 and S100A14 staining were assessed by immunoreactive score, which is the product of the intensity and percentage scores. KCNN4 and S100A14 staining pattern was defined as low (0–6) and high (8–12). The relationships of KCNN4 and S100A14 expression with clinicopathological characteristics were estimated by Chi-square tests estimated from resampled null distributions. We performed univariate and multivariate Cox regression on recurrence in our cohort to validate their independent prediction powers for recurrence. KM curves were plotted to explore their expression status in different recurrence status, and log-rank tests performed to validate their significance.

## SUPPLEMENTARY FIGURES AND TABLES








