# Supplementary material for: KCNN4 and S100A14 act as predictors of recurrence in optimally debulked patients with serous ovarian cancer
Source: Oncotarget. 2016 May 30;7(28):43924–38. doi: 10.18632/oncotarget.9721 (PMC5190068; doi:10.18632/oncotarget.9721)
Supplement: Supplementary file 1 [file oncotarget-07-43924-s001.pdf]

KCNN4 and *S100A14* act as predictors of recurrence in optimally debulked patients with serous ovarian cancer

SUPPLEMENTARY FIGURES AND TABLES

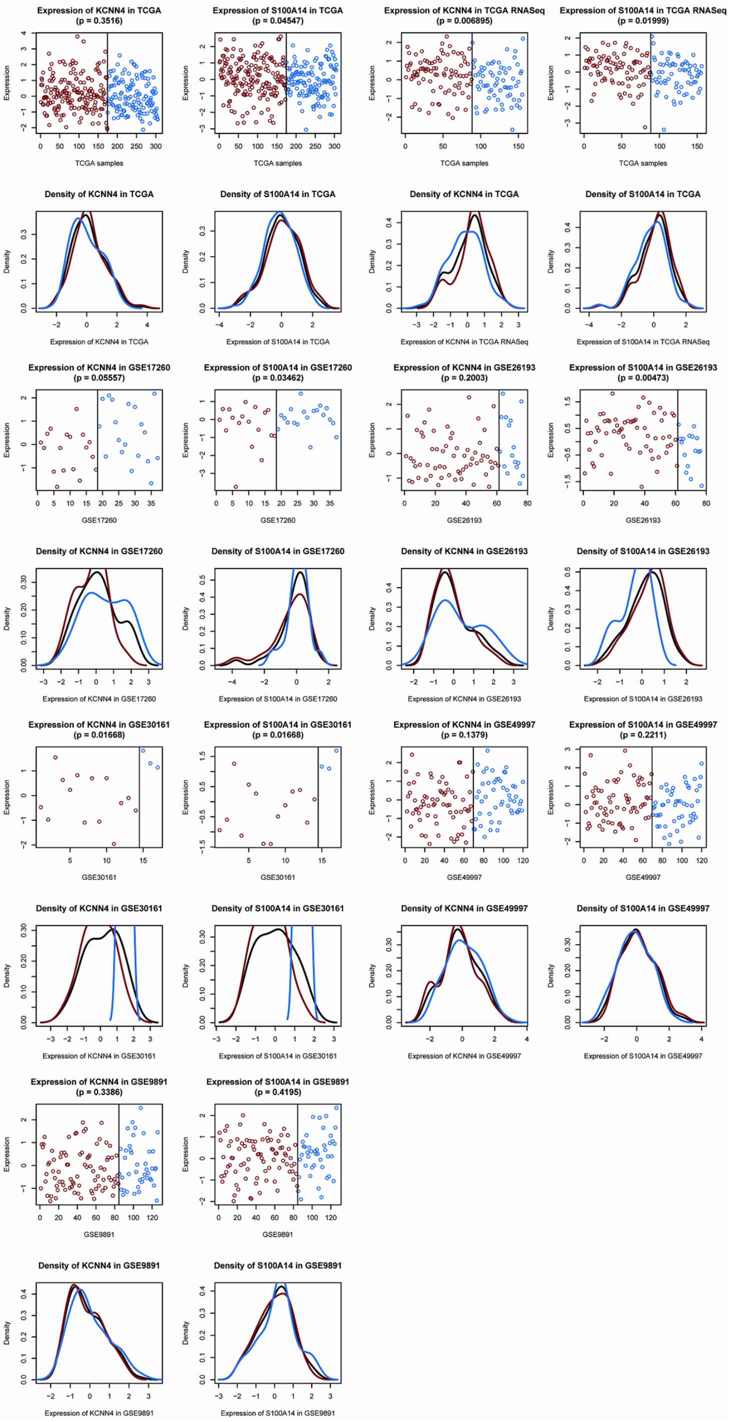

Supplementary Figure S1: Dot and density plots of *KCNN4* and *S100A14* expression in 7 public datasets. In dot plots, the red dots represent recurrence samples and the blue dots represent no recurrence samples. In density plots, the red lines represent expression distributions in recurrence samples, and the blue lines in no recurrence samples, and the black lines in all samples.

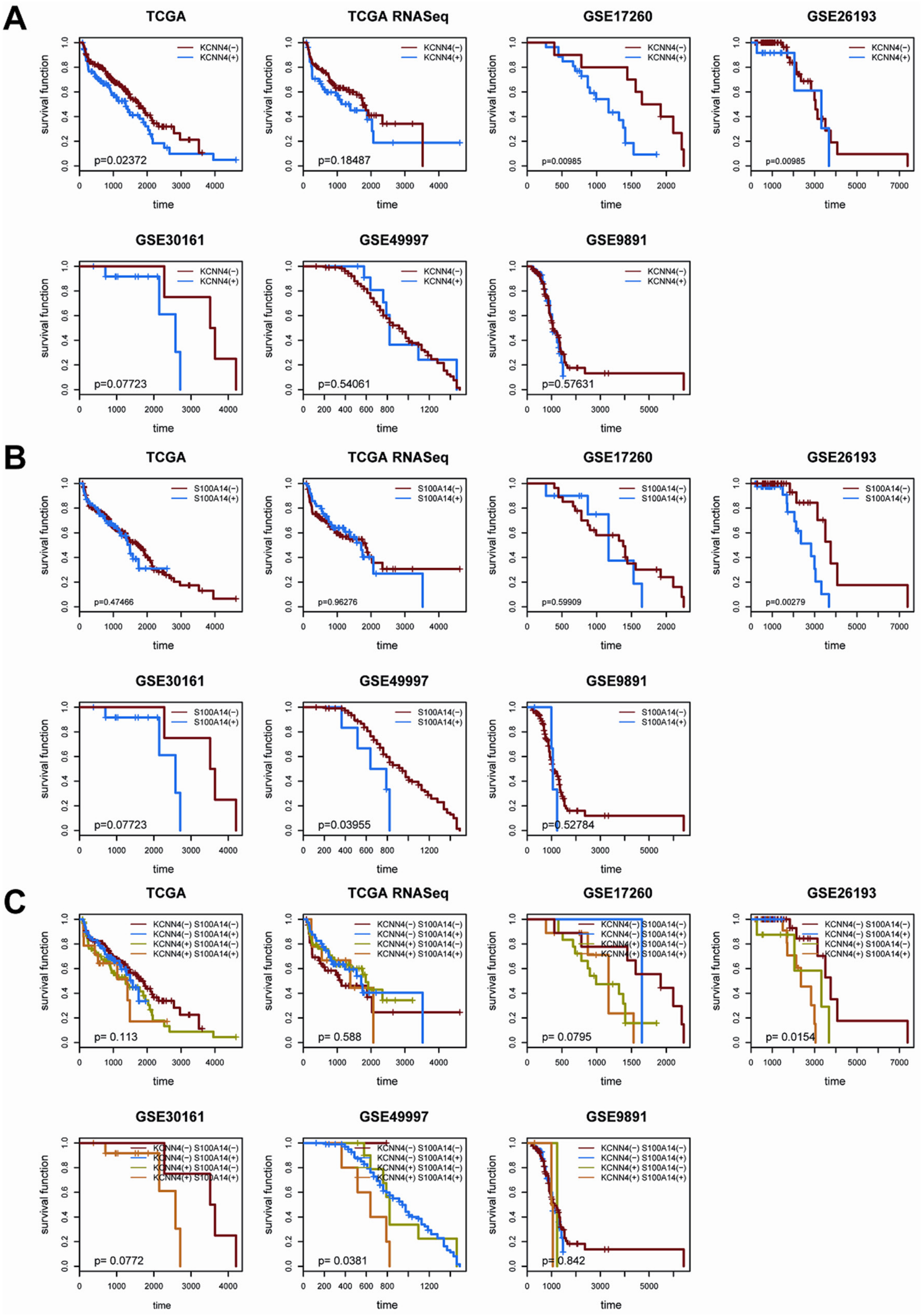

Supplementary Figure S2: Kaplan-Meier curves of *KCNN4* and *S100A14* expression status on overall survival. A. *KCNN4* expression status. **B.** *S100A14* expression status. **C.** *KCNN4* and *S100A14* expression status combinations. The p values from log-rank tests are listed.

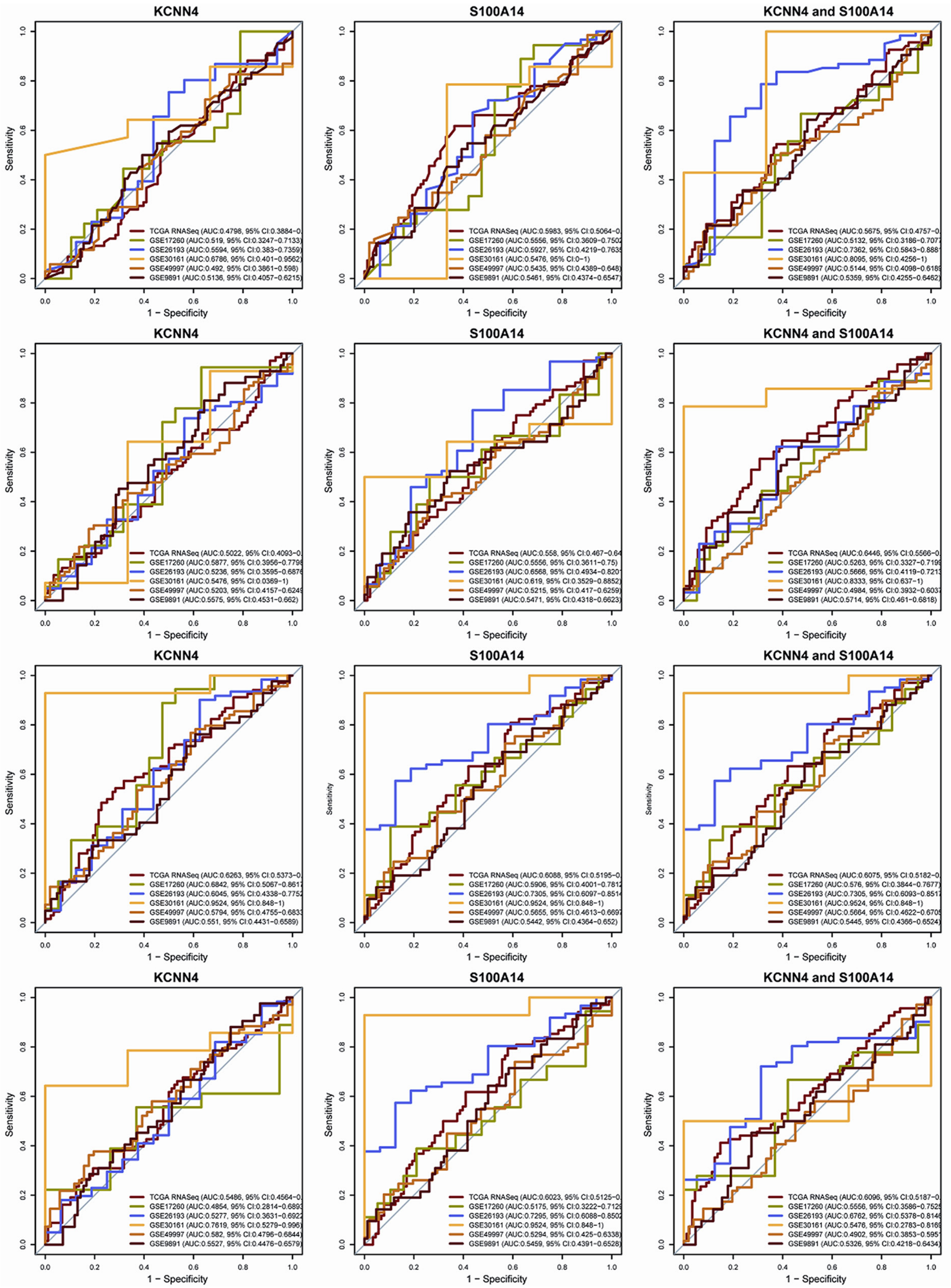

Supplementary Figure S3: The prediction power of the recurrence prediction model built in the TCGA dataset on the other 6 datasets. The models were built with **A.** random forest (rf), **B.** radial kernel SVM (svmRadial), **C.** linear kernel SVM (svmLinear), (D) artificial neuron network (nnet), via 5-repeats of 10-fold cross validation, coupled with internal parameter selection procedures. For each dataset, the AUCs and 95%CIs are also listed.

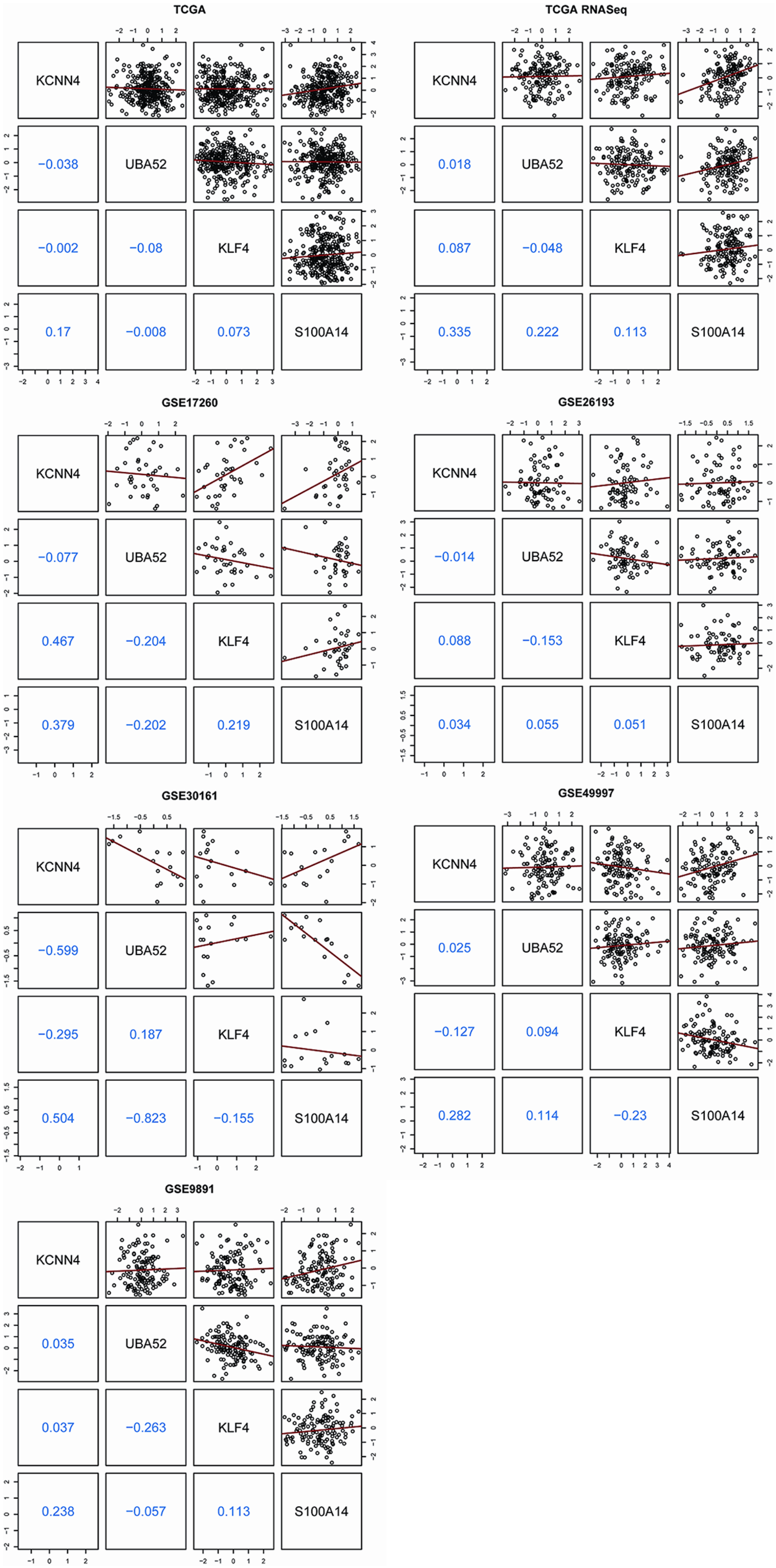

Supplementary Figure S4: pairwise expression correlations of these 4 axis genes in the 7 datasets. In the upper triangle, black dots are pairwise expression values in all samples in certain dataset, and the red lines represent linear regression. In the lower triangle, the blue figures illustrate the Pearson's correlation coefficients between either 2 genes.

Supplementary Table 1A: The relationships of clinical factors with expression status/values of *KCNN4* in 7 public datasets

Supplementary Table 1B: The relationships of clinical factors with expression status/values of *S100A14* in 7 public datasets

See Supplementary File 1A and 1B

Supplementary Table 2A: Univariate and multivariate Cox regression analysis of prognostic factors in SOC for recurrence in 7 datasets

See Supplementary File 2A

Supplementary Table 2B: Univariate and multivariate Cox regression analysis of prognostic factors in SOC for overall survival in 7 datasets

See Supplementary File 2B
