# Supplementary material for: KCNN4 and S100A14 act as predictors of recurrence in optimally debulked patients with serous ovarian cancer
Source: Oncotarget. 2016 May 30;7(28):43924–38. doi: 10.18632/oncotarget.9721 (PMC5190068; doi:10.18632/oncotarget.9721)
Supplement: Supplementary file 2 [file oncotarget-07-43924-s002.docx]

**Supplementary Table 1A the relationships of clinical factors with expression status/values of KCNN4 in 7 public datasets**

| **Clinical**  **factors** | **TCGA** | | | | **TCGA.RNASeqV2** | | | | **GSE17260** | | | | **GSE26193** | | | | **GSE30161** | | | | **GSE49997** | | | | **GSE9891** | | | |
| --- | --- | --- | --- | --- | --- | --- | --- | --- | --- | --- | --- | --- | --- | --- | --- | --- | --- | --- | --- | --- | --- | --- | --- | --- | --- | --- | --- | --- |
|  | **KCNN4 status** | | | **KCNN4**  **expression** | **KCNN4 status** | | | **KCNN4**  **expression** | **KCNN4 status** | | | **KCNN4**  **expression** | **KCNN4 status** | | | **KCNN4**  **expression** | **KCNN4 status** | | | **KCNN4**  **expression** | **KCNN4 status** | | | **KCNN4**  **expression** | **KCNN4 status** | | | **KCNN4**  **expression** |
|  | **OR** | **95% CI** | **P** | **P^c^** | **OR** | **95% CI** | **P** | **P^c^** | **OR** | **95% CI** | **P** | **P^c^** | **OR** | **95% CI** | **P** | **P^c^** | **OR** | **95% CI** | **P** | **P^c^** | **OR** | **95% CI** | **P** | **P^c^** | **OR** | **95% CI** | **P** | **P^c^** |
| **summarygrade^a^** | 0.52 | 0.21 – 1.18 | 0.11 | 0.28 | 0.51 | 0.11 – 1.74 | 0.30 | 0.89 | 0.21 | 0.02 – 1.32 | 0.07 | 0.74 | 0.54 | 0.13 – 2.45 | 0.33 | 0.17 | Inf | 0.38 - Inf | 0.24 | 0.16 | 0.21 | 0.005 – 1.54 | 0.18 | 0.21 | 1.17 | 0.52 – 2.62 | 0.71 | 0.23 |
| **summarystage^b^** | 1.06 | 0.42- 2.53 | 1 | 0.35 | 0.50 | 0.09 - 2.03 | 0.38 | 0.79 | 0 | 0 - Inf | 1 | - | 0.83 | 0.17 – 5.37 | 0.72 | 0.45 | 0 | 0 - Inf | 1 | - | 0 | 0 – 14.47 | 1 | 0.69 | 0.62 | 0.16 – 2.02 | 0.45 | 0.30 |
| **stage** | - | - | 0.71 | 0.76 | - | - | 0.68 | 0.92 | - | - | 1 | 0.25 | - | - | 0.38 | 0.67 | - | - | 1 | - | - | - | 1 | 0.79 | - | - | 0.34 | 0.61 |
| **grade** | - | - | 0.16 | 0.24 | - | - | 0.18 | 0.24 | - | - | 0.07 | 0.77 | - | - | 0.14 | 0.09 | - | - | 0.19 | 0.35 | - | - | 0.18 | 0.21 | - | - | 0.32 | **0.02** |
| **age_at_ diagnosis** | - | - | 0.50 | 0.23^d^ | - | - | 0.59 | 0.17^d^ | - | - | - | - | - | - | - | - | - | - | 0.46 | 0.50^d^ | - | - | 0.72 | 0.90^d^ | - | - | **0.03** | **0.01**^d^ |
| **pltx** | 0.79 | 0.18 – 2.84 | 0.78 | 0.86 | 1.30 | 0.32 – 4.83 | 0.76 | 0.84 | 0 | 0 - Inf | 1 | - | - | - | - | - | 0 | 0 - Inf | 1 | - | - | - | - | - | 1.42 | 0.20 – 8.85 | 0.69 | 0.92 |
| **tax** | 1.55 | 0.55 – 4.18 | 0.34 | 0.71 | 2.85 | 0.46 – 20.28 | 0.22 | 0.09 | 0 | 0 - Inf | 1 | - | - | - | - | - | Inf | 0.05 - Inf | 1 | 0.37 | - | - | - | - | 1.61 | 0.59 – 4.31 | 0.35 | 0.63 |
| **recurrence status** | 1.79 | 1.08 – 2.98 | **0.02** | 0.13 | 3.18 | 1.52 – 6.79 | **0.001** | **0.01** | 14.2 | 1.58 – 705.97 | **0.01** | 0.06 | 5.34 | 1.17 – 24.84 | **0.014** | 0.20 | Inf | 1.98 - Inf | **0.01** | **0.02** | 4.11 | 0.82 – 40.35 | 0.07 | 0.14 | 1.82 | 0.76 – 4.58 | 0.17 | 0.35 |
| **vital status** | 1.48 | 0.89 – 2.45 | 0.12 | 0.07 | 1.28 | 0.63 – 2.64 | 0.50 | 0.23 | 4.35 | 0.46 - 218.54 | 0.23 | 0.78 | 1.65 | 0.32 – 7.30 | 0.48 | 0.57 | Inf | 0.93 - Inf | **0.03** | **0.04** | 1. | 0.16 – 4.40 | 1 | 0.14 | 1.66 | 0.74 – 3.75 | 0.19 | 0.13 |
| **site of recurrence** | 0.97 | 0.48 – 1.97 | 1 | 0.72 | 1.94 | 0.61 – 6.93 | 0.30 | 0.95 | - | - | - | - | - | - | - | - | - | - | - | - | - | - | - | - | - | - | - | - |

a summarygrade: “low” represents FIGO grade I-II, “high” represents grade III-IV

b summarystage: “low” represents FIGO stage I-II, “high” represents stage III-IV

c Wilcoxon-Mann-Whitney rank sum test or Kruskal-Wallis test with exact null distribution formed via resampling

d Spearman linear correlation test

**Supplementary Table 1B the relationships of clinical factors with expression status/values of S100A14 in 7 public datasets**

| **Clinical**  **factors** | **TCGA** | | | | **TCGA.RNASeqV2** | | | | **GSE17260** | | | | **GSE26193** | | | | **GSE30161** | | | | **GSE49997** | | | | **GSE9891** | | | |
| --- | --- | --- | --- | --- | --- | --- | --- | --- | --- | --- | --- | --- | --- | --- | --- | --- | --- | --- | --- | --- | --- | --- | --- | --- | --- | --- | --- | --- |
|  | **S100A14 status** | | | **S100A14**  **expression** | **S100A14 status** | | | **S100A14**  **expression** | **S100A14 status** | | | **S100A14**  **expression** | **S100A14 status** | | | **S100A14**  **expression** | **S100A14 status** | | | **S100A14**  **expression** | **S100A14 status** | | | **S100A14**  **expression** | **KCNN4 status** | | | **S100A14**  **expression** |
|  | **OR** | **95% CI** | **P** | **P^c^** | **OR** | **95% CI** | **P^c^** | **P** | **OR** | **95% CI** | **P** | **P^c^** | **OR** | **95% CI** | **P** | **P^c^** | **OR** | **95% CI** | **P** | **P^c^** | **OR** | **95% CI** | **P** | **P^c^** | **OR** | **95% CI** | **P** | **P^c^** |
| **summarygrade^a^** | 1.16 | 0.47 – 3.29 | 0.83 | 0.14 | 1.20 | 0.37 – 4.58 | 1 | 0.47 | 1.38 | 0.26- 8.27 | 0.72 | 0.62 | 1.61 | 0.20 – 1.84 | 0.46 | 0.34 | 0 | 0 – 2.62 | 0.24 | 0.1914 | 2.65 | 0.34 – 20.88 | 0.2207 | 0.35 | 2.29 | 0.18 – 123.14 | 0.63 | 0.16 |
| **summarystage^b^** | 3.60 | 0.86 – 32.17 | 0.08 | 0.43 | 2.95 | 0.62 – 28.23 | 0.23 | 0.64 | 0 | 0 - Inf | 1 | - | 1.42 | 0.42 - 5.04 | 0.59 | 0.68 | 0 | 0 - Inf | 1 | - | 0 | 0 – 33.45 | 1 | 0.63 | 0.52 | 0.04 – 28.76 | 0.48 | **0.03** |
| **stage** | - | - | 0.23 | 0.67 | - | - | 0.31 | 0.53 | - | - | 1 | 0.84 | - | - | 0.93 | 0.92 | 0 | 0 - Inf | 1 | - | - | - | 1 | 0.83 | - | - | 0.39 | 0.06 |
| **grade** | - | - | 0.69 | 0.26 | - | - | 0.86 | 0.62 | - | - | 0.88 | 0.67 | - | - | 0.67 | 0.63 | - | - | 0.19 | 0.14 | - | - | 0.35 | **0.02** | - | - | 0.69 | 0.10 |
| **age_at_ diagnosis** | - | - | 0.07 | **0.01**^d^ | - | - | **0.01** | 0.17^d^ | - | - | - | - | - | - | - | - | - | - | 0.46 | 0.43^d^ | - | - | 0.89 | 0.39^d^ | - | - | 0.99 | **0.03**^d^ |
| **pltx** | 0.46 | 0.13 - 1.81 | 0.18 | 0.27 | 0.39 | 0.10 - 1.43 | 0.12 | 0.36 | 0 | 0 - Inf | 1 | - | - | - | - | - | 0 | 0 - Inf | 1 | - | - | - | - | - | Inf | 0.03 – Inf | 1 | 0.52 |
| **tax** | 1.14 | 0.35 – 4.83 | 1 | 0.55 | 3.05 | 0.35 – 143.73 | 0.43 | 0.17 | 0 | 0 - Inf | 1 | - | - | - | - | - | Inf | 0.05 - Inf | 1 | 0.30 | - | - | - | - | Inf | 0.16 – Inf | 0.58 | 0.53 |
| **recurrence status** | 2.12 | 1.13 – 4.13 | **0.01** | **0.045** | 2.91 | 1.33 – 6.68 | **0.005** | **0.02** | 3.28 | 0.59 – 24.07 | 0.15 | 0.35 | 9.18 | 1.86 – 90.23 | **0.002** | **0.005** | Inf | 1.98 - Inf | **0.006** | **0.017** | 7.28 | 0.78 – 353.95 | 0.08 | 0.22 | 6.28 | 0.49 – 338.42 | 0.11 | 0.42 |
| **vital status** | 1.11 | 0.61 – 2.02 | 0.78 | 0.76 | 1.14 | 0.55 - 2.36 | 0.73 | 0.76 | 2.28 | 0.35 – 14.02 | 0.41 | 0.76 | 1.82 | 0.57 – 6.30 | 0.30 | 0.39 | Inf | 0.93 - Inf | **0.03** | 0.63 | 1.70 | 0.18 – 83.40 | 1 | 0.07 | 2.0 | 0.16 – 107.78 | 1 | 0.07 |
| **site of recurrence** | 0.91 | 0.43 – 1.92 | 0.86 | 0.48 | 0.66 | 0.25 - 1.71 | 0. 39 | 0.52 | - | - | - | - | - | - | - | - | - | - | - | - | - | - | - | - | - | - | - | - |

a summarygrade: “low” represents FIGO grade I-II, “high” represents grade III-IV

b summarystage: “low” represents FIGO stage I-II, “high” represents stage III-IV

c Wilcoxon-Mann-Whitney rank sum test or Kruskal-Wallis test with exact null distribution formed via resampling

d Spearman linear correlation test
