# Supplementary material for: KCNN4 and S100A14 act as predictors of recurrence in optimally debulked patients with serous ovarian cancer
Source: Oncotarget. 2016 May 30;7(28):43924–38. doi: 10.18632/oncotarget.9721 (PMC5190068; doi:10.18632/oncotarget.9721)
Supplement: Supplementary file 3 [file oncotarget-07-43924-s003.docx]

Supplementary Table 2A univariate and multivariate Cox regression analysis of prognostic factors in SOC for recurrence in 7 datasets

| **Datasets and**  **clinical factors** | **Univariate analysis** | | | **Multivariate analysis** | | |
| --- | --- | --- | --- | --- | --- | --- |
|  | **HR** | **95%CI** | **Pr(>\|z\|)** | **HR** | **95%CI** | **Pr(>\|z\|)** |
| **TCGA** |  |  |  |  |  |  |
| Summarygrade | 0.93 | 0.62 - 1.39 | 0.712 | 3.95 | 0.50 – 31.13 | 0.1918 |
| Summarystage | 2.21 | 1.23 - 3.98 | **0.00841** | 2.34 | 0.98 – 5.59 | 0.0545 |
| Stage | 1.31 | 1.01 - 1.69 | **0.0423** | 3.51 | 0.50 – 24.52 | 0.2055 |
| Grade | 1.14 | 0.80 - 1.64 | 0.462 | 0.90 | 0.57 – 1.40 | 0.6361 |
| Age(>=60y vs <60y) | 1.08 | 0.80 – 1.46 | 0.631 | 1.19 | 0.87 – 1.63 | 0.2708 |
| Pltx | 0.89 | 0.42 – 1.89 | 0.757 | 1.06 | 0.49 – 2.30 | 0.8834 |
| Tax | 2.11 | 1.04 – 4.30 | **0.0392** | 2.06 | 1.00 – 4.24 | 0.0502 |
| KCNN4 | 1.07 | 0.76 – 1.48 | 0.709 | 1.01 | 0.72 – 1.42 | 0.9446 |
| S100A14 | 1.62 | 1.14 – 2.28 | **0.00647** | 1.45 | 1.01 – 2.07 | **0.0429** |
| **TCGA.RNASeqV2** |  |  |  |  |  |  |
| Summarygrade | 0.85 | 0.47 – 1.51 | 0.57 | 4.18 | 2.17 – 8.06 | **<2e-16** |
| Summarystage | 1.65 | 0.82 – 3.31 | 0.158 | 2.80 | 0.90 – 8.68 | 0.0747 |
| Stage | 1.12 | 0.75 – 1.67 | 0.597 | 1.31 | 0.72 – 2.38 | 0.376 |
| Grade | 1.29 | 0.71 – 2.35 | 0.398 | 0.82 | 0.39 – 1.72 | 0.5935 |
| Age(>=60y vs <60y) | 1.55 | 1.01 – 2.38 | **0.0475** | 2.28 | 1.41 – 3.69 | **0.0008** |
| Pltx | 1.02 | 0.44 – 2.34 | 0.965 | 1.21 | 0.50 – 2.90 | 0.6783 |
| Tax | 1.07 | 0.34 – 3.39 | 0.911 | 0.74 | 0.22 – 2.43 | 0.614 |
| KCNN4 | 1.60 | 0.97 – 2.64 | 0.0634 | 1.92 | 1.09 – 3.39 | **0.0236** |
| S100A14 | 1.60 | 1.05 – 2.46 | **0.0307** | 1.45 | 0.90 – 2.32 | 0.1243 |
| **GSE17260** |  |  |  |  |  |  |
| Summarygrade | 0.51 | 0.20 – 1.29 | 0.154 | 1.42 | 0.11 – 18.62 | 0.7915 |
| Stage | 2.58 | 0.58 – 11.55 | 0.216 | 1.83 | 0.37 – 9.19 | 0.4613 |
| Grade | 1.86 | 0.89 – 3.89 | 0.101 | 2.17 | 0.26 – 18.10 | 0.4730 |
| KCNN4 | 11.1 | 1.47 – 100.1 | **0.0198** | 9.09 | 1.12 – 89.9 | **0.0385** |
| S100A14 | 2.27 | 0.88 – 5.88 | 0.0889 | 2.33 | 0.83 – 6.67 | 0.1056 |
| **GSE26193** |  |  |  |  |  |  |
| Summarygrade | 0.94 | 0.53 – 1.68 | 0.844 | 4.56 | 0.42 – 49.08 | 0.210 |
| Summarystage | 3.27 | 1.58 – 6.75 | **0.00138** | 1.43 | 0.39 – 5.34 | 0.591 |
| Stage | 1.75 | 1.28 – 2.40 | **0.000531** | 3.35 | 0.39 – 28.72 | 0.270 |
| Grade | 1.28 | 0.82 – 2.00 | 0.27 | 1.44 | 0.77 – 2.67 | 0.249 |
| KCNN4 | 1.85 | 0.79 – 4.35 | 0.157 | 1.27 | 0.53 – 3.03 | 0.602 |
| S100A14 | 1.41 | 0.85 – 2.35 | 0.186 | 1.30 | 0.75 – 2.25 | 0.345 |
| **GSE30161** |  |  |  |  |  |  |
| Summarygrade | 0.43 | 0.13 – 1.45 | 0.173 | 0.63 | 0.03 – 13.88 | 0.770 |
| Grade | 2.04 | 0.75 – 5.51 | 0.162 | 0.55 | 0.05 – 6.43 | 0.633 |
| Age(>=60y vs <60y) | 1.02 | 0.31 – 3.33 | 0.792 | 0.60 | 0.13 – 2.60 | 0.495 |
| KCNN4 | 6.192e-10 | 0 - Inf | 0.999 | 4.084e-10 | 0 - Inf | 0.999 |
| S100A14 | 6.192e-10 | 0 - Inf | 0.999 | - | - | - |
| **GSE49997** |  |  |  |  |  |  |
| Summarygrade | 0.51 | 0.28 – 0.92 | **0.0248** | 0.63 | 0.34 – 1.17 | 0.1434 |
| Summarystage | 3.25 | 0.45 – 23.51 | 0.242 | 0.99 | 0.12 – 8.09 | 0.992 |
| Stage | 2.83 | 1.66 – 4.83 | **0.000131** | - | - | - |
| Grade | 1.97 | 1.09 – 3.55 | **0.0248** | 2.79 | 1.52 – 5.12 | **0.0009** |
| Age(>=60y vs <60y) | 1.41 | 0.88 – 2.26 | 0.158 | 1.04 | 0.63 – 1.72 | 0.877 |
| KCNN4 | 1.75 | 0.90 – 3.45 | 0.0976 | 1.59 | 0.79 – 3.13 | 0.189 |
| S100A14 | 3.63 | 0.50 – 26.18 | 0.201 | 3.45 | 0.47 – 25.30 | 0.224 |
| **GSE9891** |  |  |  |  |  |  |
| Summarygrade | 0.89 | 0.57 – 1.38 | 0.6 | 0.12 | 0.02 – 0.76 | **0.0242** |
| Summarystage | 4.34 | 1.75 – 10.78 | **0.00159** | 1.23 | 0.24 – 6.31 | 0.802 |
| Stage | 2.40 | 1.50 – 3.83 | **0.000268** | 0.15 | 0.03 – 0.83 | **0.0295** |
| Grade | 1.14 | 0.76 – 1.69 | 0.531 | 2.36 | 0.95 – 5.89 | 0.0649 |
| Age(>=60y vs <60y) | 0.93 | 0.60 – 1.43 | 0.729 | 0.95 | 0.61 – 1.50 | 0.8374 |
| Pltx | 5.80 | 0.81 – 41.75 | 0.0808 | 8.86 | 0.94 – 83.62 | 0.0569 |
| Tax | 1.51 | 0.83 – 2.75 | 0.179 | 1.23 | 0.63 – 2.38 | 0.5439 |
| KCNN4 | 1.64 | 1.05 – 2.56 | **0.0289** | 1.59 | 1.01 – 2.50 | **0.0467** |
| S100A14 | 3.70 | 0.04 – 1.93 | 0.191 | 4.17 | 0.56 – 33.3 | 0.1619 |
