# Supplementary material for: KCNN4 and S100A14 act as predictors of recurrence in optimally debulked patients with serous ovarian cancer
Source: Oncotarget. 2016 May 30;7(28):43924–38. doi: 10.18632/oncotarget.9721 (PMC5190068; doi:10.18632/oncotarget.9721)
Supplement: Supplementary file 4 [file oncotarget-07-43924-s004.docx]

Supplementary Table 2B univariate and multivariate Cox regression analysis of prognostic factors in SOC for overall survival in 7 datasets

| **Datasets and**  **clinical factors** | **Univariate analysis** | | | **Multivariate analysis** | | |
| --- | --- | --- | --- | --- | --- | --- |
|  | **HR** | **95%CI** | **Pr(>\|z\|)** | **HR** | **95%CI** | **Pr(>\|z\|)** |
| **TCGA** |  |  |  |  |  |  |
| Summarygrade | 0.62 | 0.37 – 1.03 | 0.065 | 0.46 | 0.09 – 2.44 | 0.3655 |
| Summarystage | 0.76 | 0.48 – 1.21 | 0.246 | 0.51 | 0.23 – 1.15 | 0.1039 |
| Stage | 0.92 | 0.70 – 1.2 | 0.526 | 0.89 | 0.22 – 3.61 | 0.8689 |
| Grade | 1.49 | 0.96 – 2.32 | 0.074 | 1.13 | 0.70 – 1.81 | 0.6172 |
| Age(>=60y vs <60y) | 0.90 | 0.65 – 1.26 | 0.548 | 0.85 | 0.60 – 1.20 | 0.3452 |
| Pltx | 3.02 | 0.75 – 12.22 | 0.121 | 3.10 | 0.76 – 12.59 | 0.1139 |
| Tax | 0.74 | 0.42 – 1.29 | 0.287 | 0.79 | 0.45 – 1.40 | 0.4232 |
| KCNN4 | 1.47 | 1.05 – 2.04 | **0.0245** | 1.47 | 1.04 – 2.08 | **0.0272** |
| S100A14 | 1.16 | 0.77 – 1.76 | 0.476 | 1.28 | 0.84 – 1.96 | 0.2534 |
| **TCGA.RNASeqV2** |  |  |  |  |  |  |
| Summarygrade | 0.73 | 0.36 – 1.47 | 0.373 | 1.162e-07 | 0 - Inf | 0.9950 |
| Summarystage | 0.90 | 0.46 – 1.76 | 0.751 | 0.86 | 0.27 – 2.73 | 0.8030 |
| Stage | 0.90 | 0.56 – 1.45 | 0.657 | 2.011e-07 | 0 - Inf | 0.9951 |
| Grade | 1.23 | 0.65 – 2.37 | 0.518 | 0.87 | 0.39 – 1.92 | 0.7232 |
| Age(>=60y vs <60y) | 1.00 | 0.62 – 1.63 | 0.986 | 1.02 | 0.62 – 1.68 | 0.9458 |
| Pltx | 3.20 | 0.78 – 13.06 | 0.106 | 3.59 | 0.87 – 14.83 | 0.0781 |
| Tax | 0.53 | 0.19 – 1.46 | 0.219 | 0.56 | 0.20 – 1.57 | 0.2722 |
| KCNN4 | 1.39 | 0.85 – 2.22 | 0.186 | 1.49 | 0.91 – 2.50 | 0.1123 |
| S100A14 | 0.99 | 0.61 – 1.61 | 0.962 | 1.10 | 0.65 – 1.86 | 0.7143 |
| **GSE17260** |  |  |  |  |  |  |
| Summarygrade | 0.96 | 0.43 – 2.16 | 0.926 | 0.34 | 0.06 – 2.00 | 0.2314 |
| Stage | 1.331e-08 | 0 - Inf | 0.998 | 5.770e-09 | 0 – Inf | 0.998 |
| Grade | 0.97 | 0.56 – 1.69 | 0.914 | 0.48 | 0.15 – 1.55 | 0.2181 |
| KCNN4 | 4.01 | 1.33 – 12.5 | **0.0129** | 5.56 | 1.72 – 16.67 | **0.00427** |
| S100A14 | 1.30 | 0.51 – 3.33 | 0.594 | 1.14 | 0.43 – 3.01 | 0.7852 |
| **GSE26193** |  |  |  |  |  |  |
| Summarygrade | 1.12 | 0.39 – 3.21 | 0.824 | 9.23 | 0.17 – 487.00 | 0.272 |
| Summarystage | 1.23 | 0.47 – 3.22 | 0.674 | 0.07 | 0.01 – 1.00 | 0.0501 |
| Stage | 1.34 | 0.79 – 2.27 | 0.275 | 1.58 | 0.14 – 18.13 | 0.7151 |
| Grade | 0.95 | 0.52 – 1.75 | 0.874 | 5.48 | 1.06 – 28.18 | **0.0420** |
| KCNN4 | 1.60 | 0.51 – 5.02 | 0.423 | 2.63 | 0.49 – 14.29 | 0.2593 |
| S100A14 | 5.03 | 1.56 – 16.67 | **0.00642** | 11.1 | 2.04 – 50.1 | **0.0052** |
| **GSE30161** |  |  |  |  |  |  |
| Summarygrade | 0.42 | 0.07 – 2.65 | 0.359 | 0.19 | 0.003 – 12.50 | 0.4339 |
| Grade | 1.98 | 0.52 – 7.44 | 0.315 | 0.97 | 0.08 – 12.47 | 0.9814 |
| Age(>=60y vs <60y) | 7.86 | 0.80 – 76.84 | 0.0763 | 14.68 | 0.73 – 295.80 | 0.0796 |
| KCNN4 | 6.25 | 0.65 – 50.5 | 0.114 | 1.89 | 0.13 – 25.9 | 0.6447 |
| S100A14 | 6.25 | 0.65 – 50.5 | 0.114 | - | - | - |
| **GSE49997** |  |  |  |  |  |  |
| Summarygrade | 0.97 | 0.62 – 1.53 | 0.908 | 0.08 | 0.51 – 1.35 | 0.4475 |
| Summarystage | 1.732 | 0.55 – 5.50 | 0.352 | 2.58 | 0.63 – 10.49 | 0.1868 |
| Stage | 1.02 | 0.63 – 1.64 | 0.942 | - | - | - |
| Grade | 1.03 | 0.65 – 1.62 | 0.908 | 0.79 | 0.41 – 1.52 | 0.4744 |
| Age(>=60y vs <60y) | 0.87 | 0.56 – 1.34 | 0.529 | 0.81 | 0.52 – 1.28 | 0.3681 |
| KCNN4 | 1.20 | 0.60 – 2.40 | 0.615 | 1.41 | 0.68 – 2.92 | 0.3523 |
| S100A14 | 2.56 | 1.02 – 6.67 | **0.0442** | 2.70 | 1.06 – 7.14 | **0.0358** |
| **GSE9891** |  |  |  |  |  |  |
| Summarygrade | 1.20 | 0.76 – 1.89 | 0.43 | 7.85 | 1.29 – 47.81 | **0.0254** |
| Summarystage | 0.81 | 0.47 – 1.42 | 0.461 | 0.62 | 0.14 – 2.79 | 0.5365 |
| Stage | 0.87 | 0.63 – 1.20 | 0.385 | 6.94 | 1.34 – 35.91 | **0.0209** |
| Grade | 1 | 0.70 – 1.44 | 0.999 | 1.02 | 0.45 – 2.29 | 0.9672 |
| Age(>=60y vs <60y) | 1.15 | 0.73 – 1.81 | 0.543 | 1.22 | 0.77 – 1.95 | 0.3970 |
| Pltx | 0.39 | 0.18 – 0.86 | **0.0194** | 0.06 | 0.02 – 0.22 | 2.56e-05 |
| Tax | 1.82 | 0.95 – 3.51 | 0.0732 | 3.99 | 1.37 – 11.57 | **0.0109** |
| KCNN4 | 0.86 | 0.52 – 1.43 | 0.571 | 0.82 | 0.48 – 1.42 | 0.4788 |
| S100A14 | 1.48 | 0.46 – 4.73 | 0.512 | 1.24 | 0.37 – 4.13 | 0.7250 |
